# Pin1 promotes GR transactivation by enhancing recruitment to target genes

**DOI:** 10.1093/nar/gkt624

**Published:** 2013-07-25

**Authors:** Toryn M. Poolman, Stuart N. Farrow, Laura Matthews, Andrew S. Loudon, David W. Ray

**Affiliations:** ^1^Centre in Endocrinology and Diabetes, Institute of Human Development, University of Manchester, Manchester, M13 9PT, UK, ^2^Respiratory Therapy Area, GSK, Stevenage, SG1 2NY, UK, ^3^Faculty of Life Sciences, University of Manchester, Manchester, M13 9PT, UK and ^4^Manchester Academic Health Sciences Centre, Manchester M13 9NT, UK

## Abstract

The glucocorticoid receptor (GR) is a ligand activated transcription factor, serving to regulate both energy metabolism and immune functions. Factors that influence cellular sensitivity to glucocorticoids (GC) are therefore of great interest. The N-terminal of the GR contains numerous potential proline-directed phosphorylation sites, some of which can regulate GR transactivation. Unrestricted proline isomerisation can be inhibited by adjacent serine phosphorylation and requires a prolyl isomerise, Pin1. Pin1 therefore determines the functional outcome of proline-directed kinases acting on the GR, as *cis*/*trans* isomers are distinct pools with different interacting proteins. We show that Pin1 mediates GR transactivation, but not GR trans-repression. Two N-terminal GR serines, S203 and S211, are targets for Pin1 potentiation of GR transactivation, establishing a direct link between Pin1 and the GR. We also demonstrate GC-activated co-recruitment of GR and Pin1 to the GILZ gene promoter. The Pin1 effect required both its WW and catalytic domains, and GR recruitment to its GRE was Pin1-dependent. Therefore, Pin1 is a selective regulator of GR transactivation, acting through N-terminal phospho-serine residues to regulate GR recruitment to its target sites in the genome. As Pin1 is dysregulated in disease states, this interaction may contribute to altered GC action in inflammatory conditions.

## INTRODUCTION

Glucocorticoids (GCs) are highly potent anti-inflammatory agents but also exert important effects on carbohydrate metabolism, resulting in off-target phenomena including diabetes and obesity. Therefore, there is considerable interest in identifying how the broad spectrum of glucocorticoid activities can be targeted, to retain the beneficial anti-inflammatory actions, but minimize metabolic off-target effects. Glucocorticoids act through the glucocorticoid receptor (GR), a member of the nuclear receptor superfamily. The GR undergoes extensive post-translational modification in response to both ligand binding and also activation of cellular stress pathways (1). The best characterized modifications lie in the N-terminal domain, and consist of proline-directed serine phosphorylation sites. For some target genes, the importance of individual modifications has been defined, e.g. transactivation of IGFBP1 requires phosphorylation of serine 211 (S211), and thereby recruitment of the co-activator MED14 (2). Some GR phosphorylation sites have been shown to enhance, for example, S211 and S203 (3), whereas others inhibit GR transactivation, for example, at S226 (4) and S404 (5) [reviewed in (6)].

Proline can adopt either a *cis* or *trans* conformation and is typically found on the solvent-accessible surface of proteins. Proline isomerisation therefore offers a molecular switch for recruitment of protein binding partners and with the slow intrinsic timescale of spontaneous isomerisation results in essentially separate ‘pools’ of isomers. Serine or threonine phosphorylation essentially prevents spontaneous isomerization at adjacent proline residues (pS/T-P), which then requires isomerization by Pin1 (7). Pin1 is only able to isomerise phosphorylated S/T-P motifs (8). This is important as both kinases and phosphatases acting on these pS/T-P sites require a *trans*-proline isomer. Therefore, the GR phosphosites at both S203 and S211 are potential Pin1 targets, raising the possibility that Pin1 is an important mediator of GR function. Interestingly, post-phosphorylation regulation of protein function now appears to be important for many cellular processes and diseases, including neurodegeneration (9), lipid metabolism (10) and activation of the toll-like receptor pathways (11). The Pin1 knockout mouse has a mild phenotype, similar to the cyclin D1 knockout (12), a phenotype predicted as Pin1 is required for stable cyclin D1 expression, through action on β-catenin (13).

Pin1 expression is augmented in both cancer (14–16) and inflammation (17), two pathophysiological states associated with altered GR function (17), and associates with other nuclear hormone receptors (NHRs) including the androgen receptor (18), estrogen receptor (19), Nur77 (20), PPARγ (21) and retinoic acid receptor (22). In each case, Pin1 binds to and regulates the stability of the receptor. In addition, the steroid co-activator protein 3 (SRC3), important for ER and PR, was also found to be partly dependent on Pin1 (23).

As Pin1 has been shown to modulate several NHRs and is regulated in both inflammation and cancer, we investigated its role in glucocorticoid action. Sequence analysis revealed a number of possible Pin1 target sequences within the GR N-terminus, suggesting a possible direct mode of action. We found that GR transactivation, but not trans-repression, was dependent on Pin1. Indeed, both GR and Pin1 were found to be recruited to target gene enhancers in response to GC, and co-immunoprecipitation revealed direct interaction between the two proteins. Targeted mutation of GR at either the S203 or S211 Pin1 sites rendered the GR independent of Pin1 action. Recruitment of GR to target enhancers was found to be Pin1 dependent, and this required an intact Pin1 WW domain, which is responsible for phospho-protein binding. As Pin1 had previously been shown to regulate SRC3 function, we determined that the target genes analysed were not regulated by SRC3. Taken together, we demonstrate a role for Pin1 in selectively regulating GR transactivation and thereby promoting recruitment to target sites in the genome to enhance transactivation. The increased expression of Pin1 in inflammation and in cancer is therefore predicted to selectively enhance GR transactivation, with consequences for cell function in that environment.

## MATERIALS AND METHODS

### Materials

Protease inhibitor cocktail (Complete Ultra), Proteinase K and PhosSTOP were from Roche. PCR purification kit was from Qiagen. siRNA against Pin1 (s10544 and s10545) and SRC-3 (s15698) were from Life Technologies and control siRNA (D-001810-03-05) and Pin1 (ON-TARGETplus SMARTpool, Pin1 L-003291-00-0005) from Dhamacon. Bradford Protein Assay (Coomassie Plus) (Thermo Scientific); Novex SDS-page kit and Reagents [4–12% bis-tris gels, Lithium dodecyl sulfate (LDS)-sample buffer], Dynal protein A/G beads, Optimem, Lipofectamine RNAiMax were from Life Tecnologies. Juglone was from Merckbiosciences. Halo-tag purification and detection kit, ReliaPrep RNA extraction kit, DNase I, protease inhibitor cocktail, GoScript and GoTaq were all from Promega. All other materials were from Sigma-Aldrich. See Supplementary Methods for further details of the antibodies and PCR primers (Eurofins MWG Operon) used in this study.

### Generation of halo-tag proteins

The GR halo-tag plasmid, pFN21AB9466 N-terminal Halo-tag vector, was purchased from Promega. Site-directed mutagenesis was performed using the Quik change kit (Agilent Technologies). See Supplementary Methods for details of PCR primers.

### Cell culture and transfections

A549 and HEK293T cells were obtained from the Health Protection Agency Culture Collection; both cell lines were grown in high glucose DMEM containing 10% foetal calf serum. siRNA transfections were carried using Lipofectomine siRNAmax with 10 nM of each siRNA in Optimem. All other transfections were carried out using Fugene 6; plasmids were used at 2.5 μg per 10 cm^3^ of cell culture area.

### Cell lysis and immunoprecipitation

Following cell treatments, A549 cells were washed twice in PBS before being resuspended into lysis buffer [modified from (24)] containing the following: 250 mM NaCl, 20 mM Bicine (pH 7.4), 1 μM CaCl_2_, 1 μM ZnCl_2_, 3 mM MgCl_2_, 0.5% IGEPAL, protease and phosphatase inhibitors. Cell extracts were DNase treated for 15 min and cleared by centrifugation. Protein quantification was carried out using the Bradford assay and BSA as a standard. Immunoprecipitations were carried out on 500 µg of total protein with 2 µg of antibody for 1 h at 4°C, immunocomplexes were captured using Protein A or G for 30 min. Complexes were washed in lysis buffer 6 times before being resuspended in 19.5 µl of lysis buffer, 3 µl of DTT (1 M) and 7.5 µl of NuPage LDS sample buffer. Seven percent of the input was used as a comparison to the immunoprecipitate sample.

### Western blotting

Western blotting was carried using the Nupage system (Life Technologies) according to the manufacturer’s instructions. Nupage MES running buffer was used throughout. Antibodies (see Supplementary Methods) were used at 1:1000 in Tris-buffered saline [150 mM NaCl and 20 mM Tris (pH 7.5)] with 0.1% Tween-20 (TBST) and 5% non-fat milk powder. Immunoblots were incubated overnight at 4°C. Following three washes in TBST, immunoblots were incubated with anti-mouse or –rabbit horseradish peroxidase (HRP)-linked secondary antibodies (used at 1:10 000) for 1 h. Following three further washes, immunoreactive bands were visualized using ECL (Thermo Scientific).

### Chromatin immunoprecipitation

Chromatin immunoprecipitation (ChIP) protocols were modified from on previously published methods (25,26). Cells (10^7^) were stimulated with 100 nM dexamethasone (DEX) for 45 min and then fixed with 11× formaldehyde (50 mM Bicine-KOH at pH 8; 1 mM EDTA; 0.5 mM EGTA; 100 mM NaCl; 11% formaldehyde) for 10 min. Cross-linking was stopped by the addition of glycine to a final concentration of 125 mM for 5 min. Cells were then washed in PBS (600× *g* for 5 min at 4°C), before 1 ml of lysis buffer (50 mM Bicine-KOH at pH 8; 1 mM EDTA; 0.5 mM EGTA; 85 mM KCl; 10% glycerol; 0.5% IGEPAL; protease and phosphatise inhibitors) was added and incubated for 10 min at 4°C. The crude nuclei were collected by centrifugation (600× *g* for 5 min at 4°C). Nuclear extracts were re-suspended in 1 ml of wash buffer (10 mM Tris–HCl at pH 8; 1 mM EDTA; 0.5 mM EGTA; 200 mM NaCl; protease inhibitor cocktail) and centrifuged at 600× *g* for 5 min. In all, 0.6 ml of RIPA buffer (10 mM Tris–HCl at pH 8; 1 mM EDTA; 0.5 mM EGTA; 140 mM NaCl; 1% Triton X-100; 0.1% sodium deoxycholate; 0.1% SDS; protease inhibitors) was added to the nuclear pellets. Samples were sonicated (using a probe sonicator at 30% power) for 30 s bursts followed by 30 s of cooling on ice for a total sonication time of 3 min per sample (producing 1000–500 bp fragments). Samples were centrifuged at 16 000× *g* for 10 min at 4°C. Two micrograms of antibody was added to cleared chromatin extract and incubated with rotation at 4°C overnight (50 µl retained for input). Samples were then centrifuged again, and supernatants were transferred to fresh tubes containing pre-washed (in RIPA) protein A or G Dynal beads (10 µl in 100 μl of RIPA buffer) and incubated for 1 h. The beads were washed twice with RIPA buffer and once with RIPA buffer containing 250 mM NaCl. Hundred microlitres of digestion buffer was added (50 mM Tris at pH 8; 1 mm EDTA; 300 mM NaCl; 0.5% SDS; 100 μg/ml proteinase K) and placed at 55°C for 3 h and then for 95°C for 15 min. Samples were purified using a PCR purification kit (eluted in 20 µl). qPCR was performed on 5 µl of each sample and expressed a percentage of the input sample (27).

### qPCR and reporter gene assays

RNA was extracted from A549 cells using the Rellia prep RNA extraction kit according to the manufacturer’s instructions. qPCR was performed using the GoScript transcription system and the GoTaq qPCR master mix. qPCR was carried out using the StepOne Plus real time PCR system and then analysed using the StepOne software V2 (using the comparative C_T_ method and Rpl19 as a house-keeping gene). See Supplementary Methods for qPCR primers. A549 cells were transfected as described earlier in the text with Pin1 siRNA, Halo-GR (WT and serine/proline mutants) and MMTV-luciferase (with Renilla-CMV to normalize for differences in transfection efficiency) in 10 cm^3^ plates. Sixteen hours later, cells were replated into 12-well plates. DEX was added 6 h before luciferase was measured.

### Halo-tag ChIP system

Halo-tag proteins were expressed as in A549 cells in 10 cm^3^ plates. Cells were serum starved for 6 h and then stimulated with DEX (100 nM) for 30 min. The media was removed, and the cells were washed twice in cold PBS. Five hundred microlitres of Halo-cytoplasmic lysis buffer [85 mM KCl, 10 mM Bicine (pH 7.4), 1% IGEPAL and protease inhibitor cocktail]. Lysates were centrifuged at 700× g for 5 min and then resuspended in 600 µl of buffer consisting of 150 mM NaCl, 10 mM Tris [pH 7.5] 1% triton ×100, 0.5% deoxycholate (w/v) and sonicated as described earlier in the text. Two hundred microlitres Halo link resin was pre-washed in TBS supplemented with 0.05% IGEPAL. Following an overnight incubation, the resin was washed in lysis buffer, high salt buffer (lysis buffer with 500 mM NaCl) and then 2 × H_2_O. Hundred microlitres of digestion buffer was added and prepared as described earlier in the text.

### Statistical analysis

Statistical analysis was performed using SPSS v16, multiple comparisons were analysed using a one-way analysis of variance (ANOVA) or univariate general linear model (GLM) with a Bonferroni post-hoc test. Non-parametric data were analysed using a Kruskal–Wallis one-way analysis of variance.

## RESULTS

### The effect of Pin1 inhibition on GR trans-activation and trans-repression

**Figure 1.**
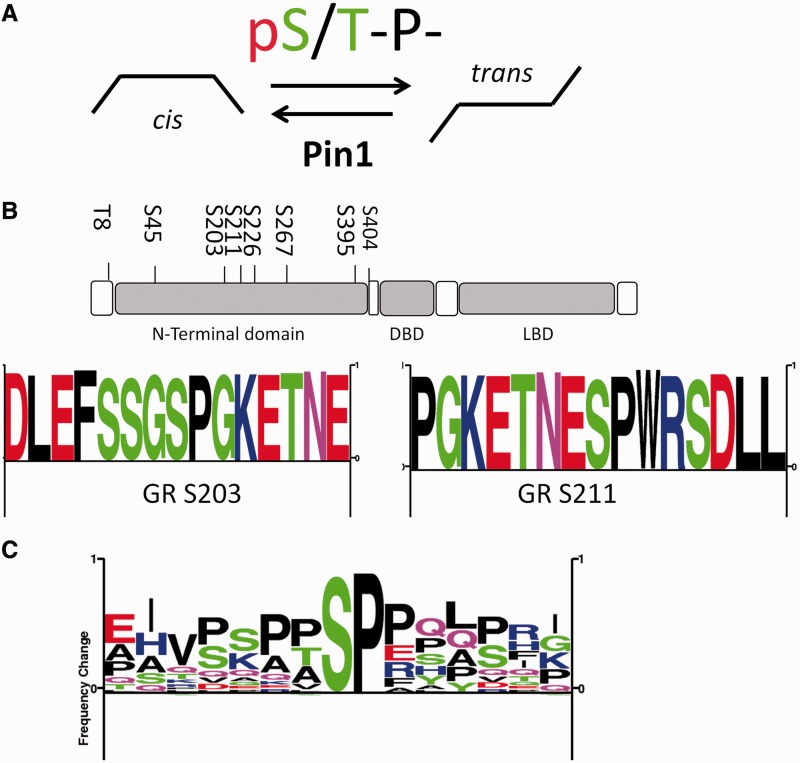
Proline-directed phosphorylation sites in the GR. (**A**) Pin1 sites binds to pS/T-P sites [e.g. WFYpSPR (8)]; *cis/trans* isomerization at pS/T-proline sites is reduced by phosphorylation (7), but is then a target for Pin1, which catalyzes *cis/trans* isomerization at pS/T-P motifs. (**B**) The major phosphosites found in the N-terminal region of GR (28). The majority of phosphosites in GR are proline-directed S/T-P sites. Some of these sites have been shown to be required for transactivation (S203 and S211), whereas some have shown to have a negative effect on GR activation (S226 and S404). Eight of the major proline-directed GR sites were used to create the sequence logo (28). (**C**) Pin1 recognition sites [taken from (28) previously found in nuclear receptors: androgen receptor (18) estrogen receptor (19), PPARγ (21), PML (29) and Nur77(TR3 (30,31)] were used to generate a sequence frequency logo (28).

Pin1 recognizes phosphorylated serine residues, which results in *cis/trans* isomerization of proline peptide bonds in a consensus flanking amino acid context (Figure 1A). A number of such candidate motifs lie within the GR N-terminal domain (Figure 1B). Pin1-binding sites have been well documented in other members of the NHR family, shown as sequence frequency logo (Figure 1C), based on peptide sequences from the androgen receptor (18), estrogen receptor (19), PPARγ (21), PML (29) and Nur77[TR3 (30,31)].

**Figure 2.**
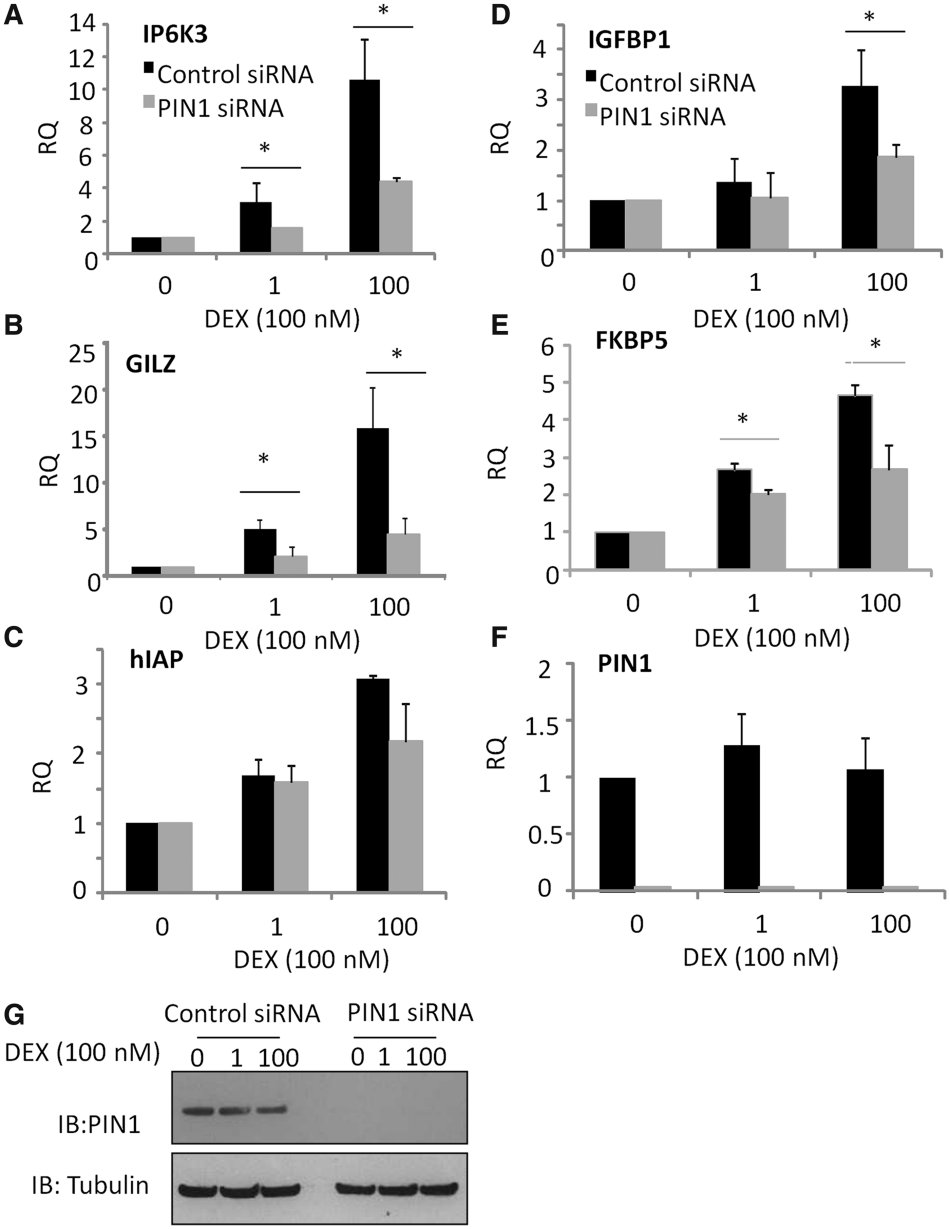
Pin1 inhibition impairs GR transactivation. A549 cells were transfected with control or Pin1 siRNA, incubated for 48 h and then stimulated with 1 or 100 nM DEX for 2 h. GC-responsive genes were measured by qPCR (**A**) IP6K3, (**B**) GILZ, (**C**) hIAP, (**D**) IGFBP1, (**E**) FKBP5. (**F**) Pin1 gene expression was also determined to demonstrate the effect of the siRNA. Graphs show mean (±SD) fold change in gene expression compared with controls (RQ). Statistical significance was determined using a one-way ANOVA with a Bonferroni post-hoc test (*P* < 0.05*) to determine the effect of Pin1 (n = 7 for GILZ and Pin1, all other genes, n = 3).

Initial studies used the Pin1 inhibitor juglone to screen for Pin1 effects on GR transactivation (Supplementary Figure S1). Juglone exerted a dose-dependent inhibitory effect on multiple GR transactivation targets, but did not affect basal expression of these genes. Pin1 knockdown was then used, and again a significant loss of transactivation (*P* < 0.05) was seen (Figure 2A–E and Supplementary Figure S2). Glucocorticoid treatment itself did not regulate Pin1 expression (Figure 2F–G).

**Figure 3.**
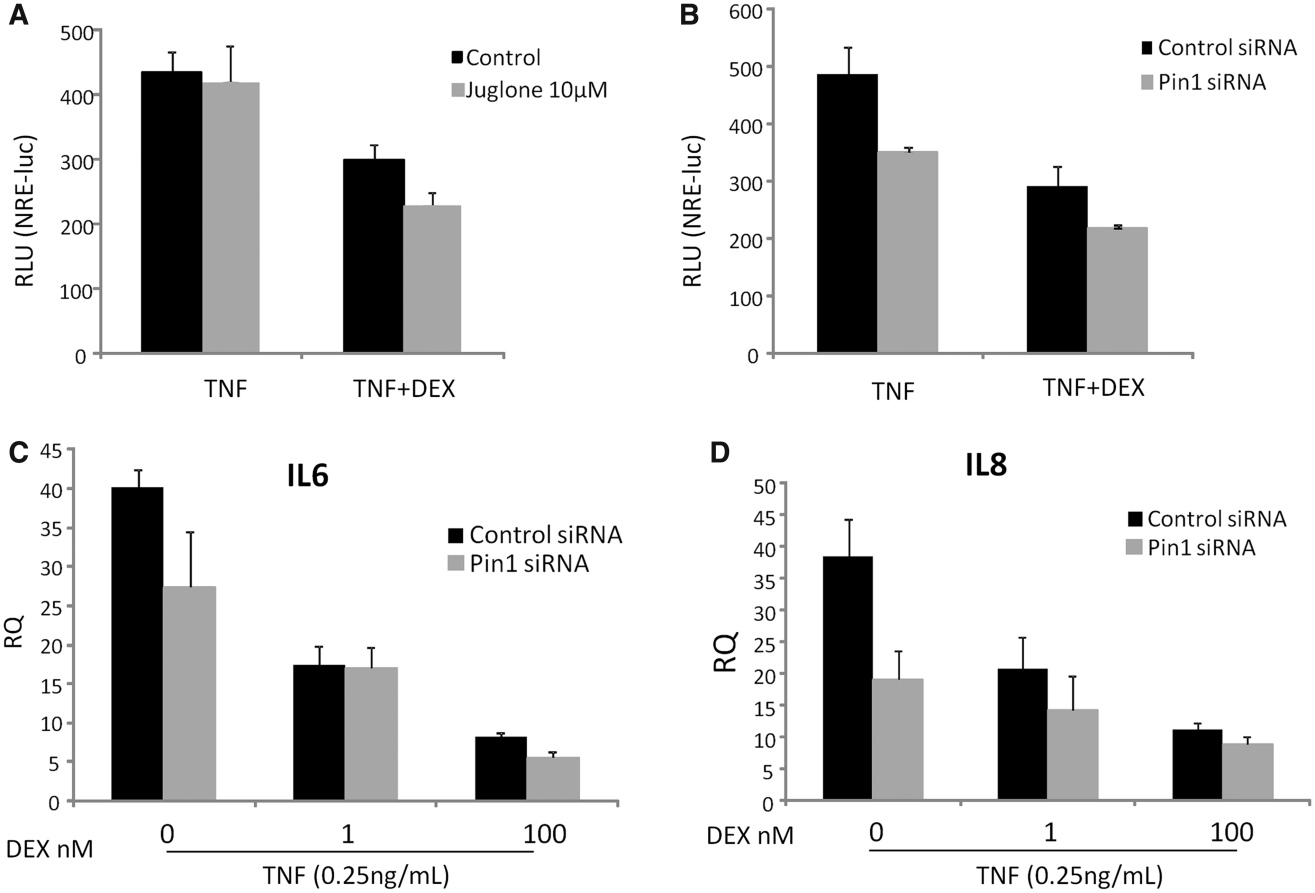
Pin1 is not required for GR trans-repression. A549 cells were treated with juglone for 1 h transfected with control or Pin1 siRNA and incubated for 48 h. Cells were then stimulated with TNFα (0.25 ng/ml) with 1 or 100 nM DEX. Cells were incubated for 16 h before the levels of NRE-luciferase (**A** and **B**) or TNFα-responsive genes (n = 3 for each) (**C**). IL-6 and (**D**). IL-8 were determined by qPCR. Graphs show mean (±SD) fold change in gene expression compared to controls (RQ). Statistical significance was determined using a GLM procedure determine the interaction between DEX and Pin1 (*P* > 0.05) on repression of TNFα-responsive genes (n = 3 for IL-6 and IL-8).

Using an NFkB reporter gene assay, the effect of Pin1 on GR-trans-represson was determined (Figure 3A). Knockdown of Pin1 expression, with siRNA, resulted in impaired tumour necrosis factor (TNF) induction of the reporter, but juglone did not show this effect, possibly reflecting cellular adaptations to prolonged loss of Pin1 protein (Figure 3A and B). However, GC inhibition was unaffected by Pin1 disruption in either case (Figure 3A and B). Analysis of the endogenous TNF target genes IL-6 and IL-8 also revealed reduced TNF induction under conditions of Pin1 knockdown, but again GC repression was unaffected (Figure 3C and D). Similar results were seen with lipopolysaccharide (LPS) induction of the cytokines IL-6 and IL-8 (Supplementary Figure S3).

### The effect of Pin1 on GR phosphorylation and stability

**Figure 4.**
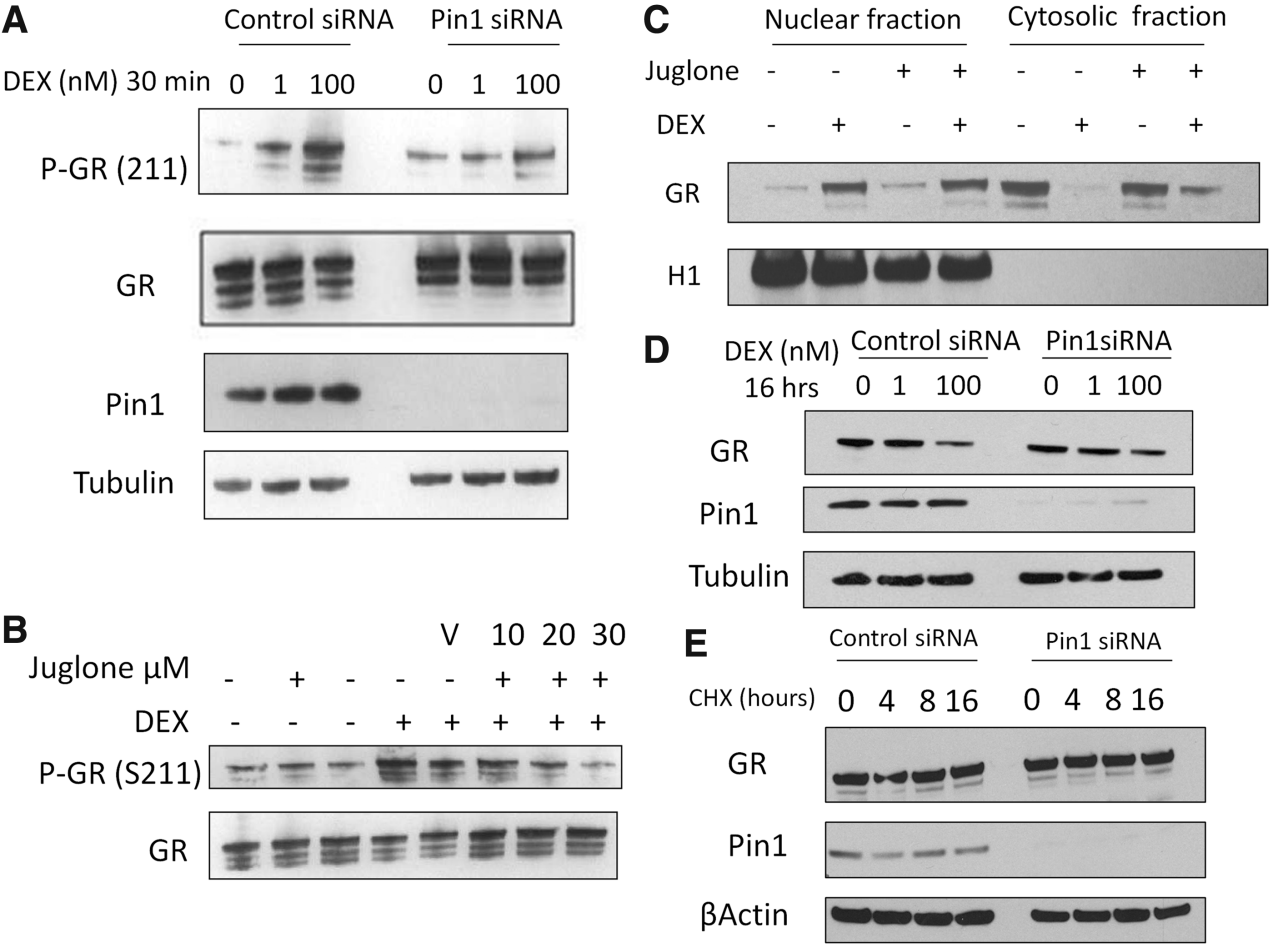
The effect of Pin1 inhibition on GR phosphorylation, nuclear translocation and stability. (**A**) A549 cells were transfected with Pin1 or control siRNA for 48 h. Cells were then treated with 1 or 100 nM for 30 min. Immunoblots were probed for phospho- GR (S211), GR, Pin1 and tubulin. (**B**) Cells were pre-treated with juglone for 30 min before a 30-min treatment of 100 nM DEX. (**C**) A549 cells were pre-treated with juglone (30 µM) for 30 min before a 30-min treatment with 100 nM DEX. Cytosol and nuclear fractions were prepared, and subsequent immunoblots were probed for GR and Histone H1. (**D**) A549 cells were transfected with Pin1 or control siRNA for 48 h before being stimulated with DEX (100 nM) or 4 or 8 h, whole-cell extracts were probed for GR and Pin1. (**E**) A549 cells were transfected with Pin1 siRNA as described in (**D**), cycloheximide (50 µg/ml) was added to the cells for 4, 8 and 16 h, subsequent immunoblots were probed for GR, Pin1 and β-actin.

**Figure 5.**
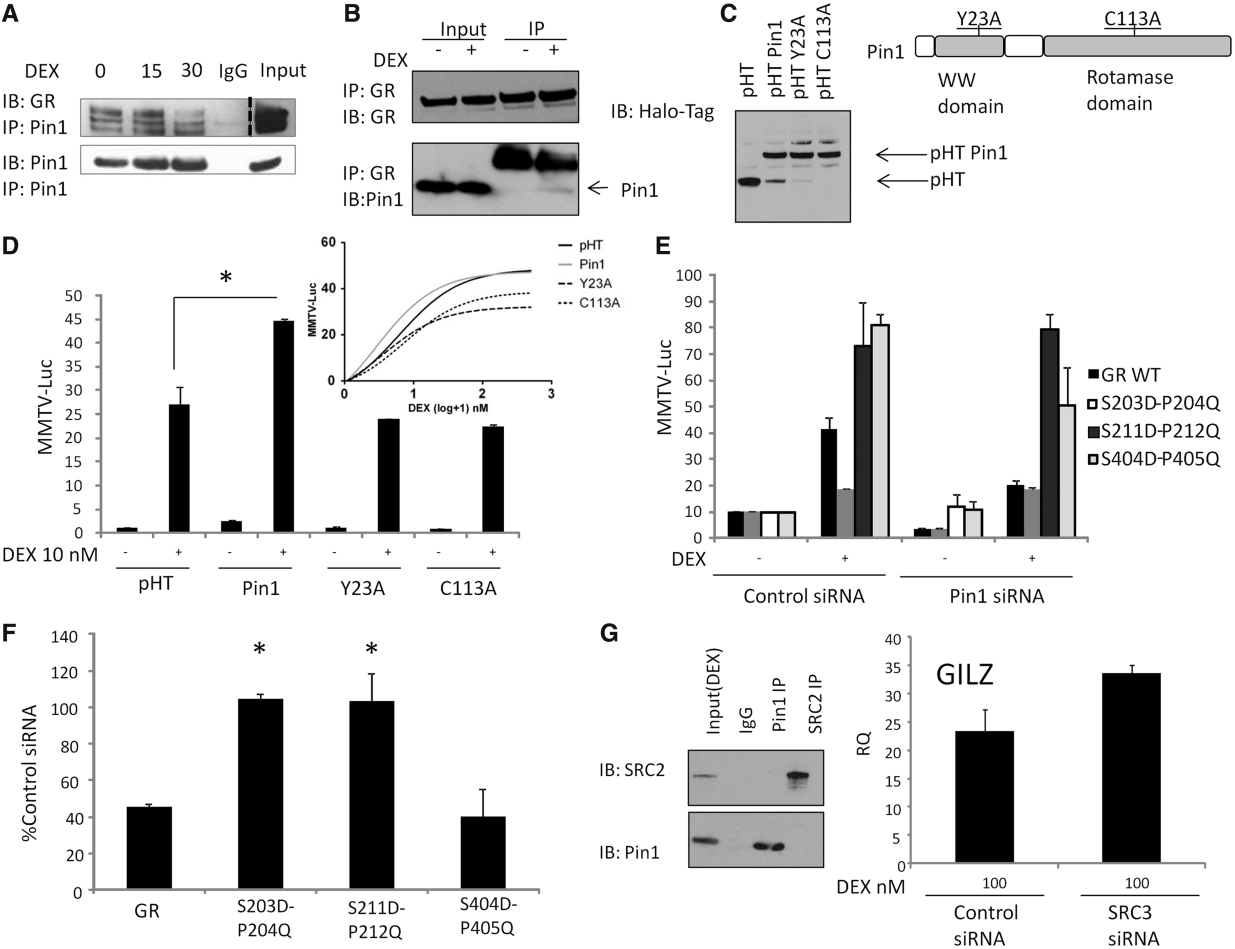
Pin1 interactions with the GR. (**A**) A549 cells were stimulated with DEX (100 nM) for the indicated time, Pin1 was immunoprecipitated from cell lysates and immunoblots probed for GR (dashed line represents a different exposure for input). (**B**) Reciprocal immunoprecipitaions for GR. Seven percent of the input sample was used in the immunoblot. (**C**) Expression of Halo-Pin1 mutants and their location in Pin1. pHalo (pHT), pHT-Pin1 WT, pHT-Pin1 Y23A and pHT-Pin1 C113A expression in A549 cells. (**D**) A549 cells were transfected with WT Pin1, Pin1 Y23A, Pin1 C113A and MMTV-luc for 16 h, after which DEX 1-500 nM was added for 6 h. (**E**) A549 cells were transfected with Pin1 siRNA for 48 h, after 24 h cells were transfected with a MMTV-luciferase reporter. In all, 10 nM DEX was added for 6 h (n = 6). (**F**) HEK293T cells were transfected with control or Pin1 siRNA for 16 h followed by transfection with MMTV-luciferase reporter and Halo-tag GR WT, S203D-P204Q, S211D-P212Q or S404D-P405Q. Cells were then pre-treated with 10 nM DEX for 6 h. Graphs show mean (±SD) of the normalized fold change in MMTV promoter activity (n = 3). (**G**) A549 cells were treated with DEX for 1 h, cell lysates were prepared and SRC-2 or Pin1 immunoprecipitated and immunobloted with either SRC-2 or Pin1 antibodies. A549 cells were treated with SRC-3 siRNA for 48 h and treated with DEX for 2 h before the levels of the GILZ gene were determined by qPCR (n = 3). Statistical analysis was determined using a one-way ANOVA and a Bonferroni post-hoc test (*P* < 0.05*).

We observed loss of the characteristic rapid GR-S211 phosphorylation in response to GC activation both with Pin1 siRNA, and also antagonism with juglone (Figure 4A and B), suggesting a role for Pin1 in acquiring or maintaining the phospho mark. Translocation of the GR to the nucleus, a critical regulatory step in gene regulation, was unaffected by Pin1 knockdown (Figure 4C), and there was no discernible effect on GR protein abundance, either under basal conditions or following ligand activation (Figure 4D). Attempts to discern differences in GR protein stability in the absence of Pin1, analogous to that seen for cyclin D1, did not reveal any significant differences (Figure 4E and Supplementary Figure S4). GR and Pin1 were also found in the same molecular complex as demonstrated using a co-immunoprecipitation immunoblot (Figure 5A and B). The strength of the interaction appeared to be enhanced by ligand activation, as demonstrated in the GR immunoprecipitation (Figure 5B).

The Pin1 domains required for recognising phosphorylated residues, or catalysing isomerisation were then targeted, by mutation to Y23A (in the WW-domain) or C113A (in the catalytic domain) (Figure 5C and D). Both of these mutations have been shown to hinder Pin1 binding to target substrates (21,32,33). Wild-type and mutant Pin1 molecules were all expressed at a similar level (Figure 5C), but neither Y23A, nor C113A were able to potentiate GR transactivation (Figure 5D).

To prove a functional effect of Pin1 directly on the GR, rather than through intermediary proteins, two GR mutants were constructed each of which contained a phosphomimetic aspartate at 203, or 211, and a glutamine in place of the adjacent proline (S203D-P204Q and S211D-P212Q). Mutations to these residues result in a phosphomimetic GR, but one not recognised by Pin1, as previously demonstrated (21). These mutant GR molecules showed variable effects on ligand-induced transactivation, with S203D-P204Q attenuated, and S211D-P212Q enhanced, but more importantly, both were unaffected by loss of Pin1 expression (Figure 5E and F). As an additional control, we mutated GR S404 (S404D-P405Q). GR S404D-P405Q showed enhanced transactivation but retained Pin1 regulation (Figure 5E and F).

### Pin1 effects on GR transactivation do not require SRC-3

Previous reports have shown that Pin1 can act on the nuclear receptor co-activator SRC-3 (23). Although we showed that the Pin1 effect on GR was dependent on phosphoserines in the N-terminal domain, and not the site of interaction between GR and the SRC family of co-activators in the C-terminal domain, additional studies were focussed on a potential role for SRC-3.

GR functions have been shown to be independent of SRC-3, whereas SRC-2 is an important co-regulator (34). Moreover, when we examined the role of SRC-3 in mediating transactivation by GR, we found no effect on our principle index gene GILZ (Figure 5G and Supplementary Figure S5 for siRNA knockdown of SRC-3). Although GR and SRC-2 interact (35), there was no interaction seen between Pin 1 and SRC-2 (Figure 5G).

### Pin1 on GR recruitment to the promoter DEX-responsive genes

**Figure 6.**
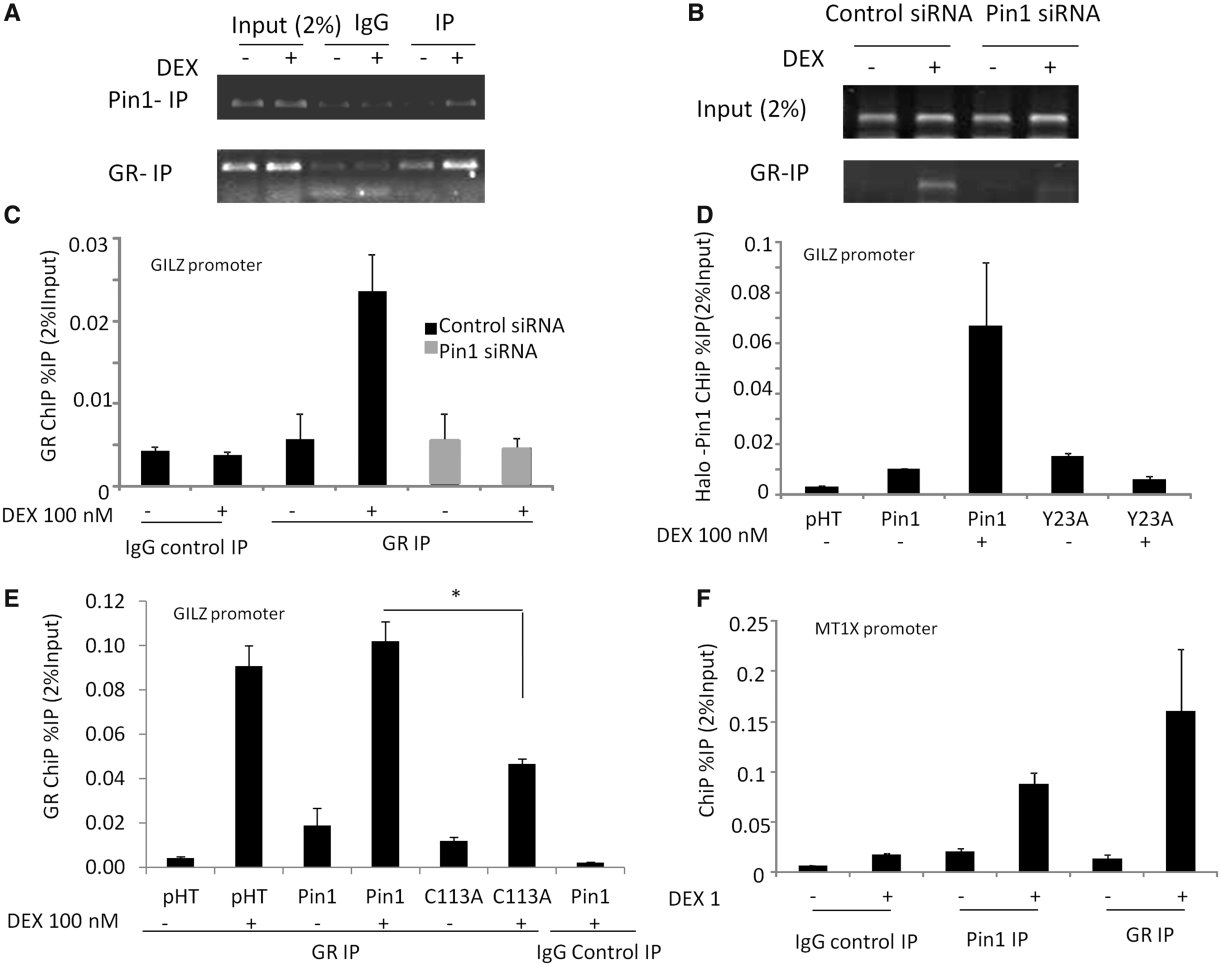
The effect of Pin1 on GR recruitment to the GILZ promoter. A549 cells were stimulated with 100 nM DEX for 1 h and chromatin prepared as described in the ‘Materials and Methods’ section, ChIP was carried out using an anti-Pin1 (**A**) or anti-GR antibody (**B**) and PCR primers for the GILZ promoter (n = 6). (**C**) A549cells were transfected for 48 h with control or Pin1 siRNA, following a 1 h DEX treatment, ChIP was carried out with an anti-GR antibody and PCR primers for the GILZ promoter. (**D**) Halo-ChIP was carried out with Halo- Control protein (pHT), Halo-Pin1 or a WW-domain mutant Pin1 (Y23A). A549 cells were transfected with each Halo tag protein for 16 h and then treated with DEX (100 nM) for 1 h before cross-linking and binding to the Halo-link resin. Pin1 loading on the GILZ promoter was determined using qPCR Results are expressed as percentage immunoprecipitaion from 2% of the input chromatin [%IP (2% input)]. (**E**) A549 cells were transfected for 16 h with control pHT, pHT-Pin1 or pHT-Pin1 C113A inactive mutant, following a 1 h DEX treatment, ChIP was carried out with an anti-GR antibody and PCR primers for the GILZ promoter (as described earlier in the text). (**F**) ChIP was carried out using as described earlier in the text using an anti-Pin1 or anti-GR antibody and PCR primers for the MT1X promoter. Statistical analysis was determined using a one-way ANOVA and a Bonferroni post-hoc test (*P* < 0.05*).

The data aforementioned suggested a specific loss of GR transactivation function that was dependent on Pin1 action on the proline-directed serine phosphorylation sites at GR S203 and S211. Using the GILZ GRE as a well-validated target for GR binding, ChIP analysis revealed GC induction of GR recruitment, as expected (Figure 6A). In addition, there was induction of Pin1 binding to the same sequence in response to GC (Figure 6A). Knockdown of Pin1 dramatically attenuated GR recruitment (Figure 6B, C). We also determined effects of Pin1 siRNA on the levels of H3K9 acetylation at the GILZ promoter (Supplementary Figure S6), showing a significant reduction. To determine whether the Pin1 effect on GR recruitment was dependent on the Pin1 WW domain, which recognizes the phosphorylated serine residues, we used the point mutated Pin1 (Y23A) characterized earlier in the text (Figure 5C and D). The mutant Pin1 was not recruited to the GILZ promoter, in contrast to wild-type Pin1 (Figure 6D). Using the Pin1 C113A, which disrupts the catalytic domain and provides a dominant negative effect, we also see an inhibitory effect on GR recruitment to the GILZ GRE (Figure 6E). To confirm these effects are seen at other GR binding sites, we analysed the MTIX gene GRE and found similar enrichment of Pin1 (Figure 6F) and GR-dependence for GR transactivation (Supplementary Figure S2).

## DISCUSSION

The GR has a broad spectrum of activities, regulating energy metabolism, and also the immune response. The ligand activated GR can both transactivate and trans-repress target gene expression, and though multiple mechanisms have been described, the precise explanation of how the same molecule can exert opposing effects on gene expression remains unclear (36). In addition, a major puzzle is how cells and tissues can vary their sensitivity to glucocorticoids, despite the near ubiquitous expression of the GR.

In particular, acquired resistance to the action of GCs in inflammation is common, with a number of contributing factors proposed, including cross-talk from other pro-inflammatory signalling pathways, for example, mediated by TNF and interferon (35) or histone deacetylase enzymes (37,38). GR is subjected to numerous post-translational modifications, including phosphorylation (39), ubiquitination (40) and sumoylation (41), some of which have been shown to contribute to resistance states (42). The majority of the phosphorylation sites on GR are proline-directed (S/T-P), which opens up the possibility that the prolyl-isomerase, Pin1, could regulate GR function. Furthermore, Pin1 expression is upregulated in inflammation and is reported to influence pro-inflammatory signalling (11). The phenotype of the Pin1 knockout mouse is complex, with premature aging, tauopathy and neurodegeneration [reviewed in (43,44)], insulin resistance (45) and also increased sensitivity to LPS challenge (46).

The GR contains a number of candidate Pin1 recognition sequences in its N-terminal domain, two of which, S203 and S211, have previously been shown to be induced by ligand binding and to be required for maximal transactivation on some DNA templates. Initial screening with the Pin1 inhibitor juglone revealed a major loss of GR transactivation, but negligible impact on GR trans-repression. This dissociation of GR function was of interest, as many of the beneficial anti-inflammatory actions of GCs come at the price of off-target gene transactivation (47). However, the non-specific effects of juglone are well documented, including effects on RNA polymerase II (48–51). Therefore, siRNA was optimized for knockdown of Pin1 protein in subsequent analyses. These revealed, again, a profound loss of GR transactivation but little impact on trans-repression.

Previous reports have determined which regulatory surface of the GR molecule is required for specific gene activation. For example, hIAP is thought to be dependent on an intact N-terminal domain, as is IGFBP1 (which is also requires the recruitment of MED14 complexes). Other genes, such as GILZ or IP6K3 are thought to be independent of the N-terminal domain (52), which includes the proline-directed phospho-serine sites. However, we discovered that both GILZ and IP6K3 were sensitive to Pin1 inhibition. This suggests that Pin1 may be acting in a way that permits both N- and C-terminal transactivation domains to be operational, such as affecting GR recruitment to its GRE sites in the genome. In contrast, GR trans-repression of NFkB driven genes is classically thought to occur through a tethering mechanism, and we did not detect any effect of Pin1 on this mode of action. SRC-2 is a co-modulator protein for GR that both activates and represses target genes (36,53); however, we did not find Pin1bound to SRC-2, in contrast to the previously reported association between Pin1 and SRC-3 (23).

As Pin1 has been shown to regulate the stability of some target substrate proteins, both in a positive and negative direction, we studied Pin1 effects on GR expression, stability, trafficking and phosphorylation. Essentially, there was no discernible effect of Pin1 loss on any of these aspects of GR, but consistent decreases in ligand activated S211 phosphorylation were observed. As S211 phosphorylation is required for maximal transactivation of some (IGFBP1), but not all (IP6K3) target genes, this was of interest. However, as the detection of S211 phosphorylation required an antibody, we could not exclude the possibility that loss of Pin1 exerted an effect on the *cis/trans* isomer balance and thus affected a conformational epitope. The kinases responsible for ligand-activated phosporylation of S203 and S211 remain obscure, but evidence has been presented for both cyclin-dependent kinases (CDKs) and mitogen-activated protein kinases (MAPKs). Such kinases require the *trans* isomer to be an effective substrate, and the balance between *cis* and *trans* is determined by Pin1 action. Therefore, Pin1 regulates the outcome of the kinase(s) acting at these two serines.

As previous reports had revealed Pin1 isomerization of SRC-3 as important for regulating ER transactivation, we sought evidence that the Pin1 effect was targeted to the GR itself. First, we were able to show that SRC-3 was not required for GR transactivation of the GILZ gene, although Pin1 was required. Second, we showed that targeted mutations to the GR, to prevent S203 and S211 phosphosite recognition by Pin1, resulted in competent GR transactivation, but were now independent of Pin1 effect. Taken together, these data support a role for Pin1 acting on the GR to modify its function, strengthened by finding Pin1/GR interaction in co-immunoprecipitation studies.

Further analysis of the Pin1 GR interaction revealed that disruption to the Pin1 proline-directed serine recognition site (WW domain) abolished the co-activation effect. We showed that ligand-activated GR binding to the well-characterised GILZ gene GRE was accompanied by recruitment of Pin1, and reciprocally, that loss of Pin1 attenuated GR binding. This suggested a role for Pin1-directed GR isomerization in promoting GR interaction with the genome, specifically to sites required for GR transactivation (36). To investigate this in more detail, we showed that mutations to the Pin1 WW domain, which blocked GR co-activation, also result in loss of Pin1 recruitment to the GRE, excluding a scaffold effect, and identifying a requirement for phospho-serine recognition. A further mutation to Pin1 (C113A), which disrupts the isomerase catalytic domain, was also shown to inhibit GR recruitment to the GRE, strengthening the mechanistic link requiring Pin1 action on the GR to permit efficient DNA binding.

Taken together, we have identified a master-regulator role for Pin1 on GR signalling. Pin1 is required for full transactivation, but not trans-repression of NFkB-activated genes. In contrast to the actions of Pin1 on other nuclear receptors, we have not found effects on GR stability or a requirement for modification of the SRC-3 co-activator to explain altered GR activity. However, we did find evidence for GR-Pin1 binding, both at the protein–protein level, and also in later studies using ChIP binding for both proteins to a GRE element upstream of the GILZ gene. We show a role for Pin1 in regulating GR binding to its GRE, thereby offering an explanation for the requirement of Pin1 for maximal GR transactivation. The Pin1 effect required an intact WW domain, required for binding phosphorylated serines, and also proline-directed serine phosphorylation of the GR. As Pin1 is regulated in inflammatory and malignant disease, we predict a selective alteration in GR function would be a consequence, as has recently been defined (36). Moreover, targeting the Pin1 effect on GR may offer a means to focus GR activity towards anti-inflammatory circuits and away from effects on energy mobilization.

## SUPPLEMENTARY DATA

Supplementary Data are available at NAR Online.

## FUNDING

DWR was funded by the Wellcome Trust (to D.W.R.); NIHR Manchester Biomedical Research Centre, and the NIHR Manchester BRU in musculoskeletal diseases; Wellcome Trust Institutional Strategic Support Fund (ISSF) award [097820] (in part) to the University of Manchester. Funding for open access charge: This work was supported by the NIHR Manchester Biomedical Research Centre, and the NIHR Manchester BRU in musculoskeletal diseases.

*Conflict of interest statement.* None declared.

## Supplementary Material

Supplementary Data
