# Supplementary material for: Pin1 promotes GR transactivation by enhancing recruitment to target genes
Source: Nucleic Acids Res. 2013 Jul 25;41(18):8515–25. doi: 10.1093/nar/gkt624 (PMC3794586; doi:10.1093/nar/gkt624)
Supplement: Supplementary Data [file supp_41_18_8515__index.html]

Pin1 promotes GR transactivation by enhancing recruitment to target genes — Pin1 promotes GR transactivation by enhancing recruitment to target genes — Supplementary Data 

# Pin1 promotes GR transactivation by enhancing recruitment to target genes

## 

files

**Files in this Data Supplement:**

- Supplementary Data - docx file
