# Supplementary material for: Pin1 promotes GR transactivation by enhancing recruitment to target genes
Source: Nucleic Acids Res. 2013 Jul 25;41(18):8515–25. doi: 10.1093/nar/gkt624 (PMC3794586; doi:10.1093/nar/gkt624)
Supplement: Supplementary Data [file supp_gkt624_nar-00986-v-2013-File008.docx]

**Supplementary information**

**Materials and Methods**

**Antibodies**

**GR western blots:** Mouse monoclonal clone 41 [BD Biosciences], Sigma Prestige <http://www.sigmaaldrich.com/catalog/product/sigma/hpa004248?lang=en&region=GB> and Novus Biologicals <http://www.novusbio.com/Glucocorticoid-Receptor-Antibody_NB300-610.html> ) **GR ChIP**: (Protein Tech <http://www.ptglab.com/Products/NR3C1-Antibody-24050-1-AP.htm> ), **Pin1** (Santa Cruz <http://www.scbt.com/datasheet-81533-pin1-8c10-antibody.htm> l and Proteintech <http://www.ptglab.com/Products/PIN1-Antibody-10495-1-AP.htm> ), **SRC2** (BD Biosciences), **Phospho-serine 211 GR antibody** (Cell Signaling) **Tubulin and Histone H1** (Proteintech <http://www.ptglab.com/Products/TUBA1B-Antibody-11224-1-AP.htm> and <http://www.ptglab.com/Products/H1F0-Antibody-17510-1-AP.htm> ). **Anti-acetylated histone antibody (H3K9)** <http://www.cellsignal.com/products/9649.html>

**PCR primers**

**GILZ** For: AATGCGGCCACGGATG Rev: GGACTTCACGTTTCAGTGGACA

**HIAP** For: GACAGGAGTTCATCCGTCAAG Rev: TTCCACGGCAGCATTAATC

**IP6K3** For: TTCTCGCTGGTGGAAGACAC Rev: CAGCAACAAGAACCGATGC

**FKBP5** For: AGGCTGCAAGACTGCAGATC Rev: CTTGCCCATTGCTTTATTGG

**IGFBP1** For: TTTCTCAAACTGCAGCCTCC Rev: TGGGCACTTCCTACAGTTCC

**MT1X** For: GAT CGG GAA CTC CTG CTT CT Rev: CTT GTC TGA CGT CCC TTT GC

**Pin1** For CGGCAGGAGAAGATCACC Rev CCTCCTCTCCCTGACTTGAT

**SCR3** For ACA ACCAGA TCC AGC CTT TGG TC Rev TGG ATG CAG CCT GCG GGT GTT GC

**IL6** For GGTACATCCTCGACGGCATCT Rev- GTGCCTCTTTGCTGCTTTCAC

**IL8** For ATGACTTCCAAGCTGGCCGTGGCT Rev TCTCAGCCCTCTTCAAAAACTTCTC

**Rpl19** For: ATGTATCACAGCCTGTACCTG Rev: TTCTTGGTCTCTTCCTCCTTG

**ChIP primers**

GILZ For: GGGAATTCTGATACCAGTTAAGC Rev:GGAGACAATAATGATCTCAGGA

MTX1 For: GCAGGTGCTCTTTGTGATGA Rev: CCCATTTGATCCCTACATGG

**HaloTag primers**

**Halo-Pin1** For –CACCGCGATCGCCATGGCGGACGAGGAGAAGCTGC Rev

TGTCGTTTAAACCTCAGTGCGGAGGATGATGTGGA

**Mutagenesis primers**

**Y23A** For  CGC AGC TCA GGC CGA GTG GCC TAC TTC AAC CAC ATC AC

Rev  GTG ATG TGG TTG AAG TAG GCC ACT CGG CCT GAG CTG CG

**C113A** For CTC ACA GTT CAG CGA CGC CAG CTC AGC CAA GGC C Rev  GGC CTT

GGC TGA GCT GGC GTC GCT GAA CTG TGA G

**GR S211D P212Q** For  GAG ACG AAT GAG GAT CAA TGG AGA TCA GAC CTG

 Rev CAG GTC TGA TCT CCA TTG ATC CTC ATT CGT CTC

**GR S404DP405Q** For GAC CAG ATG TAA GCG ATC AAC CAT CCA GCT CC Rev

GGA GCT GGA TGG TTG ATC GCT TAC ATC TGG TC

**Supplementary figures**

**Fig S1**


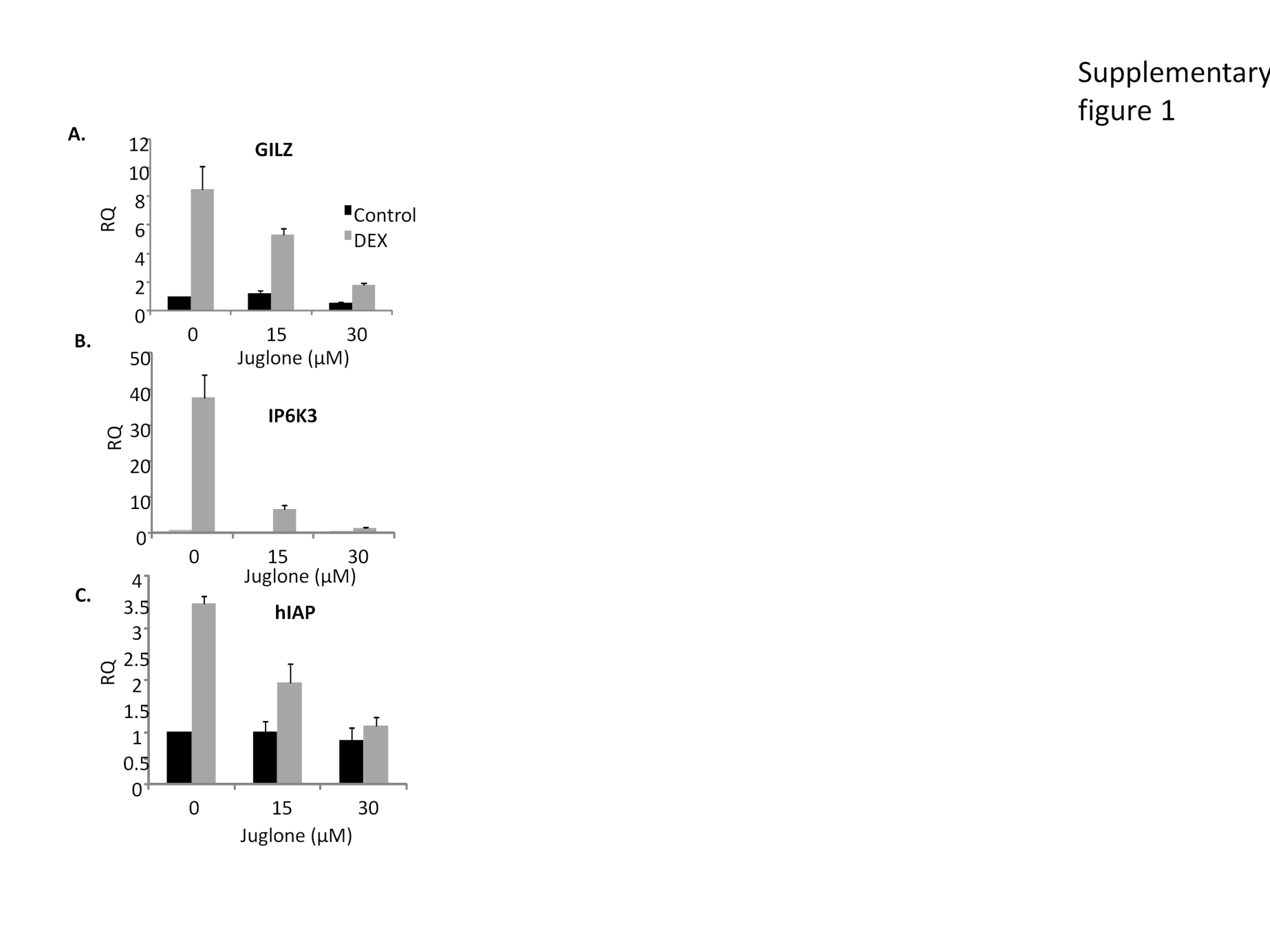


**Figure S1. Juglone inhibits GC regulated genes**. A549 cells were treated with juglone (µM) for 30 min before being stimulated with 100 nM DEX for 2 hours, the expression of GC-regulated genes A. GILZ, B. IP6K3 and C. hIAP. Gene expression was measured by qPCR. Graphs show mean (+/- SD) fold change in gene expression compared to controls (RQ). Statistical significance was determined using a general linear model to determine the effect of juglone (n=3 *p*<0.05, using a general linear model [GLM]).

**Fig S2**

**
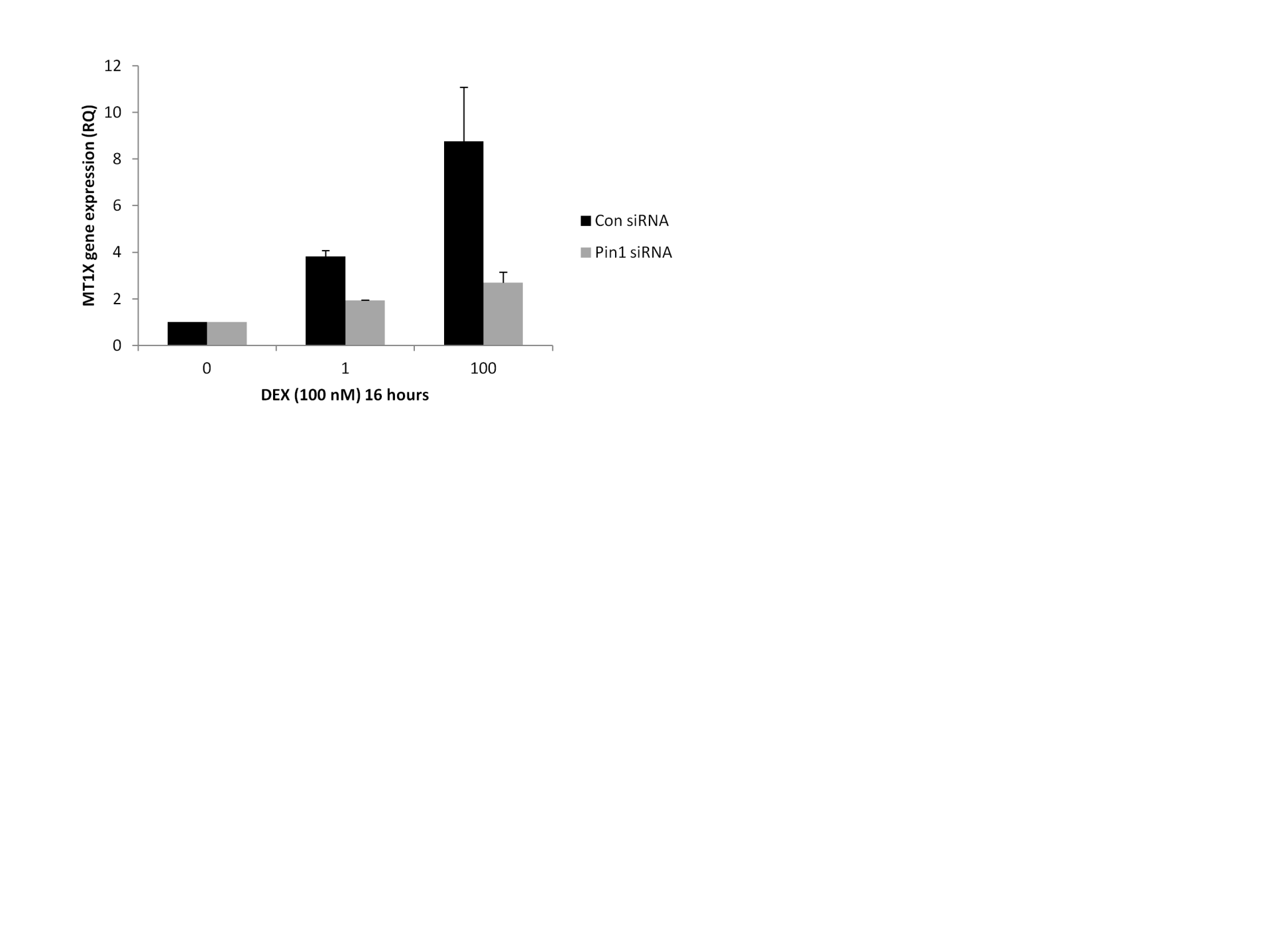
**

**Figure S2. Pin1 inhibition impairs GR transactivation of the MT1X gene**. A549 cells were transfected with control or Pin1 siRNA, incubated for 48 hours and then stimulated with 1 or 100 nM DEX for 16 hours. MT1X gene expression was measured by qPCR. Graphs show mean (+/- SD) fold change in DEX –induced gene expression compared to controls (RQ).

**
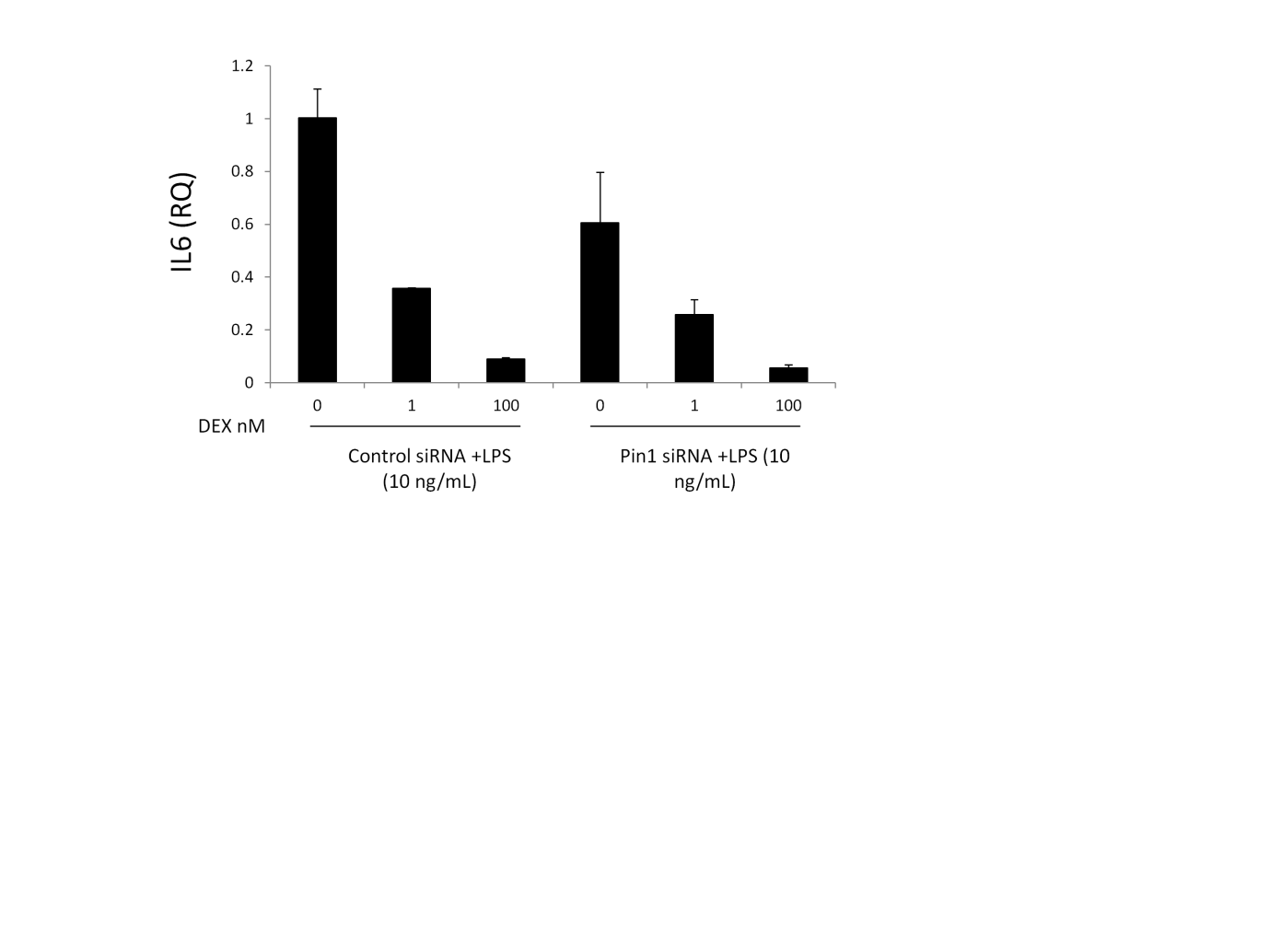
Fig S3**

**Figure S3. Pin1 is not required for GR transrepression** A549 cells were transfected with control or Pin1 siRNA, incubated for 48 hours and then stimulated with LPS (1 ng/mL) with 1 or 100 nM DEX. Cells were incubated for 16 hours before the levels of or LPS-induced IL6 expression wasdetermined by qPCR. Graphs show mean (+/- SD) fold change in gene expression compared to controls (RQ).

**Figure S4**

**
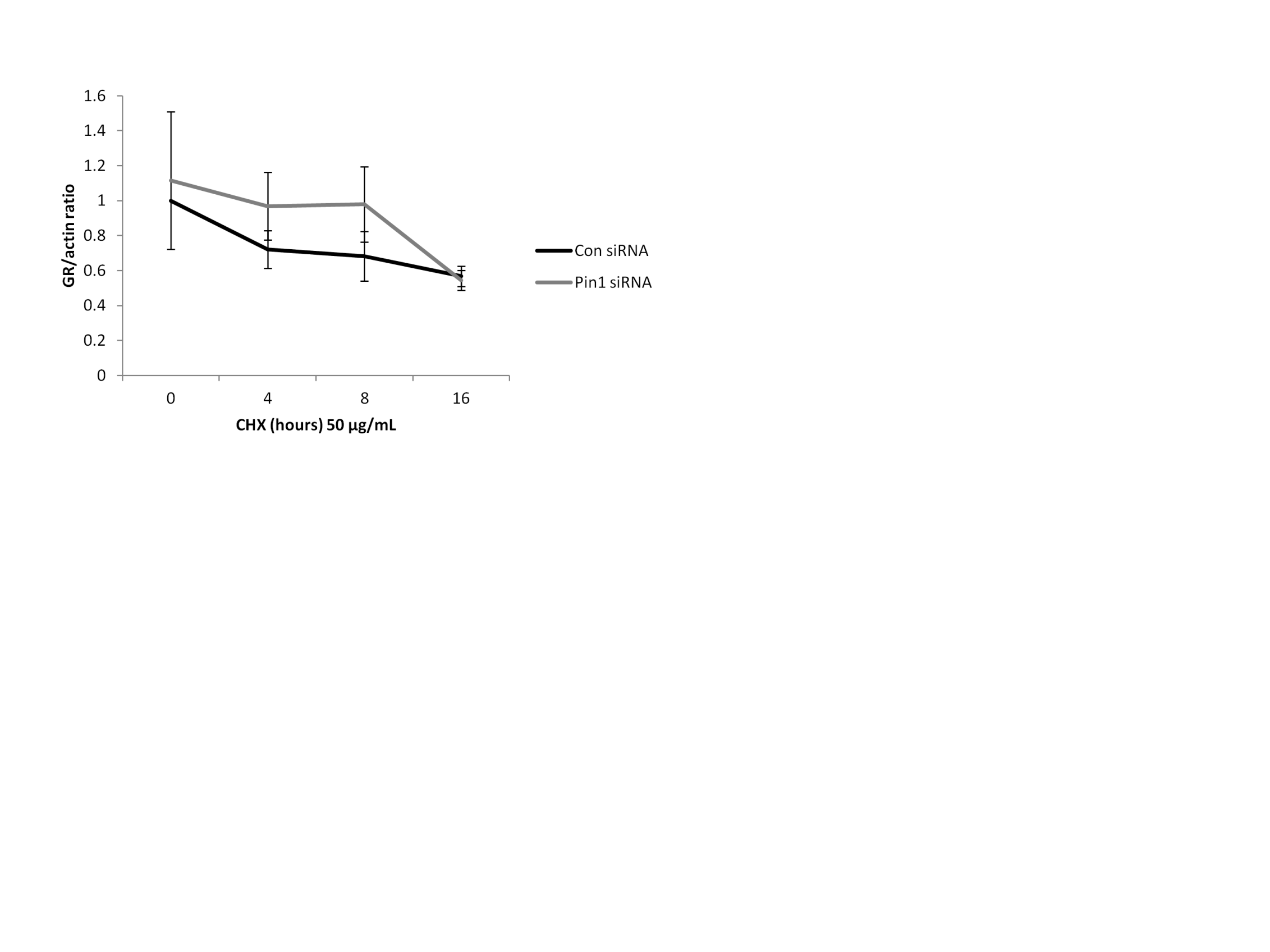
**

**Figure S4. Densitometry for Figure 4E.** A549 cells were transfected with Pin1 or control siRNA for 48 hours. Cycloheximide (CHX) (50 µg/mL) was added to the cells for 4, 8 and 16 hours. Subsequent immunoblots were probed for GR and β-actin. Immunoblots were analysed using Image J software (<http://rsbweb.nih.gov/ij/index.html>). Graphs show mean (+/- SD) GR/β-actin ratios. Statistical significance was determined using a non-parametric Kruskal–Wallis one-way analysis of variance (*p*>0.05).

**Figure S5**


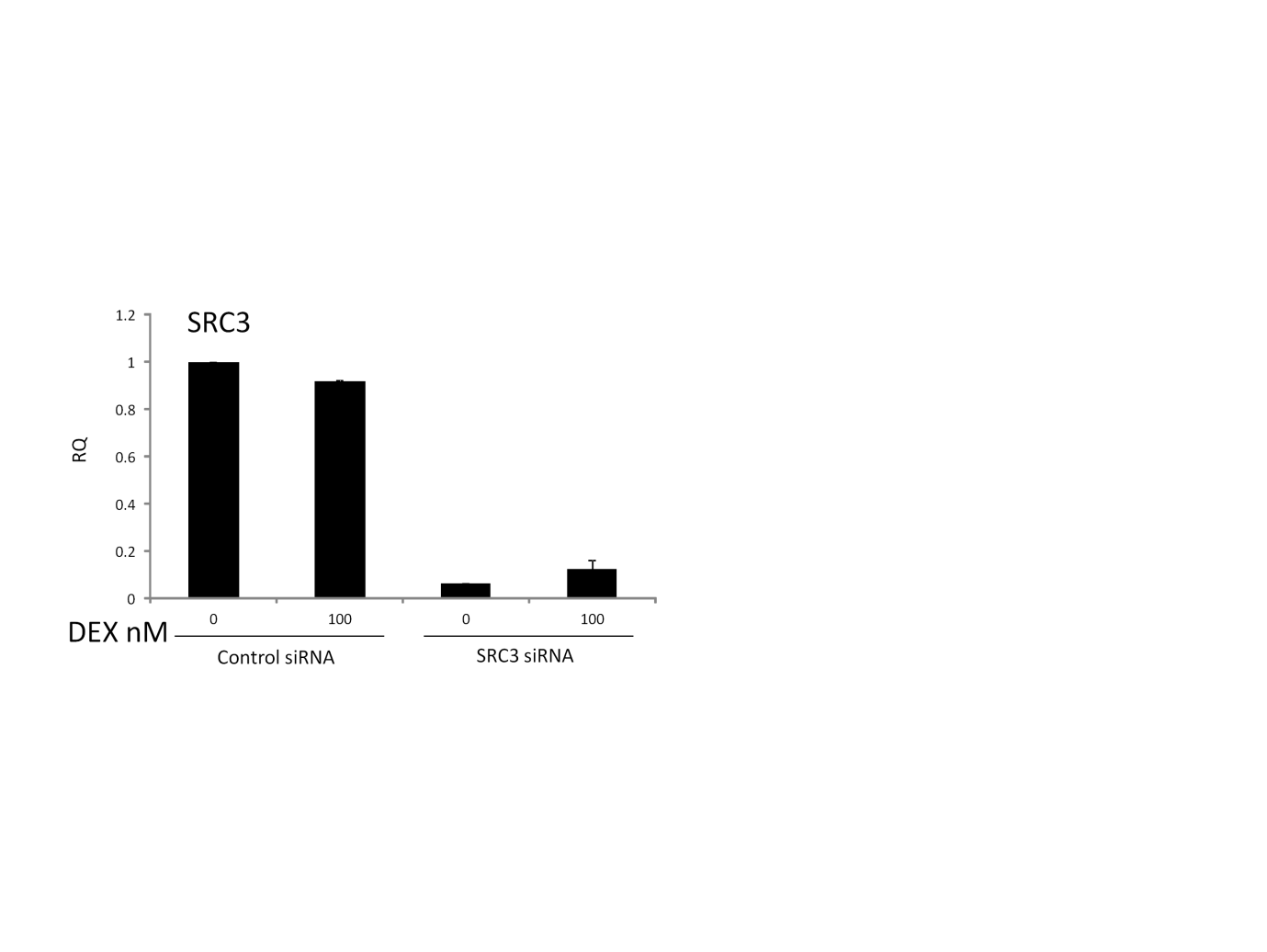


**Figure S5**. A549 cells were transfected with control or SRC-3 siRNA, incubated for 48 hours and then stimulated with 1 or 100 nM DEX for 16 hours. SRC-3 gene expression was determined by qPCR. Graphs show mean (+/- SD) fold change in gene expression compared to controls (RQ).

**Figure S6**


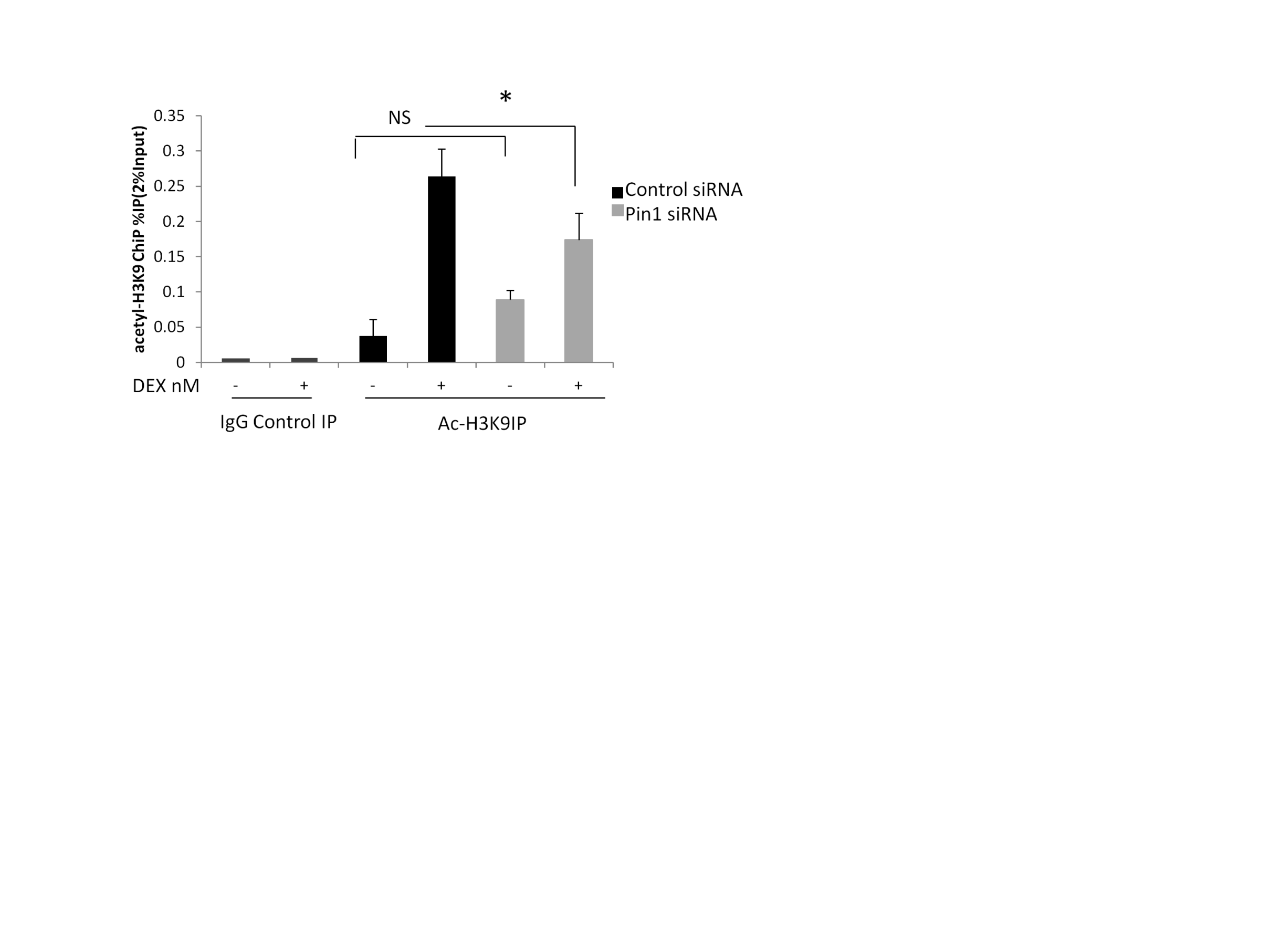


**Figure S6**. **Pin1 inhibition antagonises DEX-induced H3K9 acetylation.** A549 cells were transfected for 48 hours with control or Pin1 siRNA, following a 1 hour DEX treatment ChIP was carried out with an anti-GR antibody and PCR primers for the GILZ promoter as described in the materials and methods. Statistical significance was determined using a one-way ANOVA and Bonferroni post-hoc test (*, *p*<0.05).
